# Total Hip Arthroplasty for Untreated Acetabular Fractures: A Case Series

**DOI:** 10.1155/cro/2582466

**Published:** 2026-04-15

**Authors:** Christian Emmanuel M. Fontanilla, John N. Hermosisima, Kenneth Alexis M. Yap, Phillipe Y. Baclig

**Affiliations:** ^1^ Department of Orthopaedics and Traumatology, Vicente Sotto Memorial Medical Center, Cebu City, Cebu, Philippines

**Keywords:** acetabular fracture, hip dislocation, total hip arthroplasty

## Abstract

**Introduction:**

Acetabular fractures are managed acutely with internal fixation but when left untreated for more than 3 weeks, these injuries lead to post‐traumatic arthritis and osteonecrosis of the hip. Such complications indicate the need for total hip arthroplasty. In the Philippines, no studies have been published documenting the outcomes of managing untreated acetabular fractures with total hip arthroplasty.

**Case Presentation:**

This study documented five cases of untreated acetabular fractures managed with total hip arthroplasty in Vicente Sotto Memorial Medical Center, Philippines in 2023. The cases included four males and one female ages 21–46 years old. All cases resulted from motorcycle crashes and most presented with untreated posterior wall fractures with posterosuperior, segmental, acetabular defects, and chronic posterior hip dislocations. The chronicity of the fractures ranged from 17 to 32.5 weeks. The cases were managed with acetabular augments when needed, dual mobility cups, and femoral short stems. In the short‐term postoperative period, all cases had improved Forgotten Joint Scores and Harris Hip Scores with no incidence of infection, dislocation, or implant failure.

**Conclusion:**

Untreated acetabular fractures managed with total hip arthroplasty prevent post‐traumatic arthritis and osteonecrosis of the hip. Segmental acetabular defects and chronic hip dislocations present in these cases can be managed with acetabular augments and dual mobility cups. When these injuries present in the young, femoral short stems can be used to preserve the femoral neck and maximize the proximal metaphyseal bone stock.

## 1. Introduction

### 1.1. Background of the Study

Acetabular fractures are uncommon traumatic injuries which can be sustained from high‐energy motor vehicular crashes. Within the past two decades, their incidence has been decreasing in the United States but the opposite has been observed in Asia where motorcycle transportation is predominant [1].

In an acute setting, acetabular fractures are treated with internal fixation. In developing countries, especially in rural areas, treatment is sometimes delayed due to socioeconomic factors and limited access to healthcare facilities. When acetabular fractures have been untreated for more than 3 weeks, the hip becomes arthritic, and the femoral head undergoes osteonecrosis [2]. Total hip arthroplasty is indicated to address these complications [3–5]. This surgery also allows the earliest return to function of the patient, preventing sequelae of prolonged immobilization such as deep venous thrombosis, pressure sores, and pneumonia [5]. The Forgotten Joint Score (FJS) and Harris Hip Score (HHS) are used to quantify the improvement in the patient′s functionality after surgery.

### 1.2. Significance of the Study

In a review of recent literature, only one study in Southeast Asia has documented its outcomes in managing untreated acetabular fractures with total hip arthroplasty. This was published in Indonesia where six cases were encountered within a span of 3 years [6]. No similar studies have been published in the Philippines. This information was yielded by searching the keywords “untreated acetabular fractures” OR “neglected acetabular fractures” OR “chronic acetabular fractures” in PubMed and HERDIN wherein 167 and 0 results were generated, respectively.

### 1.3. Research Question

What are the postoperative outcomes of untreated acetabular fractures managed with total hip arthroplasty?

### 1.4. General Objectives

The aim of this study is to document the cases of untreated acetabular fractures managed with total hip arthroplasty in Vicente Sotto Memorial Medical Center (VSMMC) in 2023.

### 1.5. Specific Objectives

This study is aimed at reporting the surgical management of untreated acetabular fractures and their postoperative outcomes in terms of (a) infection, (b) dislocation, (c) implant failure, and (d) functional score (FJS and HHS).

### 1.6. Conceptual Framework

All cases of untreated acetabular fractures admitted in VSMMC in 2023 were included in this study with no excluded cases. These cases underwent total hip arthroplasty in 2023 and monitoring of postoperative outcomes was started within the same year. This case series underwent evaluation and approval by the institutional ethics review board of VSMMC.

## 2. Case Presentation

In 2023, five cases were admitted in VSMMC, Philippines for untreated acetabular fractures. Each case sustained the injury from a motorcycle crash. All cases presented with displaced fractures of the acetabulum, post‐traumatic arthritis, osteonecrosis of the femoral head, and chronic hip dislocation. The demographic data of the cases are listed in Table 1.

**Table 1:** Demographic data.

Case	Age	Sex	MOI^a^	Laterality	Fracture	Hip dislocation	Time to THA^b^
1	21	Male	Motorcycle crash	Right	Posterior wall	Posterior	31.1 weeks
2	39	Male	Motorcycle crash	Left	Posterior wall	Posterior	32.5 weeks
3	46	Male	Motorcycle crash	Right	Posterior wall	Posterior	18.4 weeks
4	22	Female	Motorcycle crash	Right	Posterior wall	Posterior	17 weeks
5	32	Male	Motorcycle crash	Right	Posterior wall	Posterior	21.9 weeks

^a^MOI: mechanism of injury.

^b^THA: total hip arthroplasty.

The cases included four males and one female with a mean age of 32 years old and a mean time from injury to arthroplasty of 24.2 weeks.

### 2.1. Case 1

**Figure 1:**
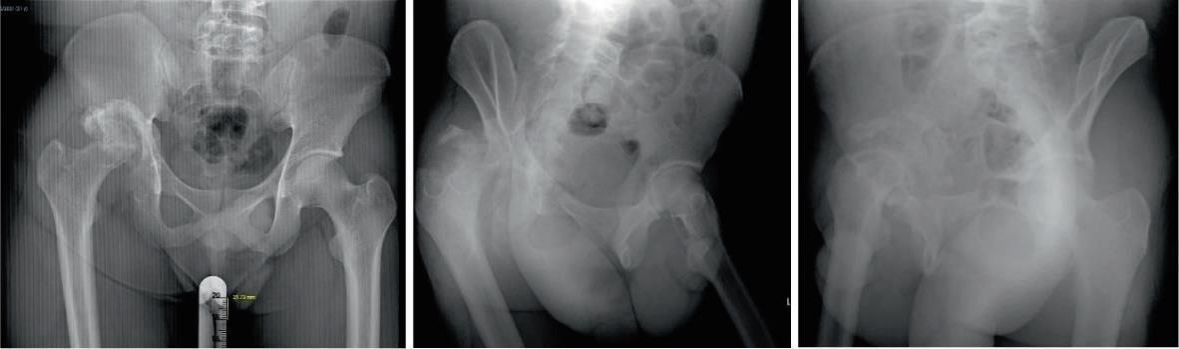
Case 1 preoperative radiographs.

**Figure 2:**
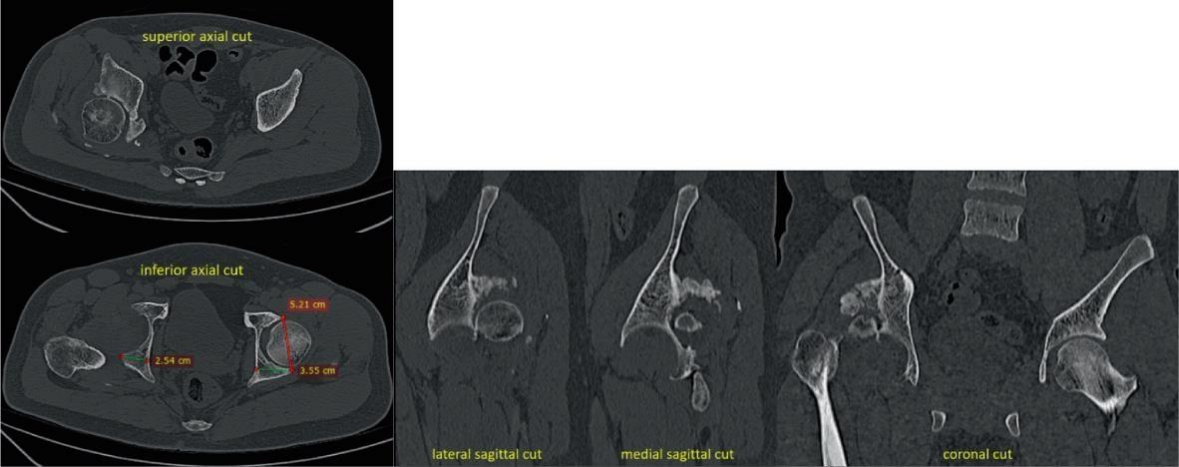
Case 1 preoperative computed tomography.

The first case was a 21‐year‐old male with an untreated acetabular fracture of the right posterior wall which presented with a segmental posterosuperior acetabular defect due to osteonecrosis of the comminuted fragments. The defect was about 1 cm deep and involved an estimated 20% of the acetabular rim, classifying the defect as Paprosky IIIa. There was an associated chronic posterior hip dislocation wherein the center of rotation (COR) had migrated proximally by 2 cm and had created a pseudo‐acetabulum, classifying the dislocation as Thompson and Epstein III. These findings are shown in Figures 1 and 2. The injuries had been untreated for 31.1 weeks prior to total hip arthroplasty. Preoperatively, the FJS was 43 and the HHS was 46. The high FJS and low HHS signify poor functional status.

The hip was exposed through a Hardinge approach and the femoral head and neck were osteotomized to serve as the structural bone graft to fill the pseudo‐acetabulum. A 54 × 10 − mm, titanium, trabecular, cup augment was used to fill the acetabular defect and a 52‐mm, cementless, dual mobility cup was employed to decrease the risk of postoperative hip dislocation. The femoral component was composed of a Size 2, cementless, short stem, and a 22/0 cobalt–chromium–molybdenum (CoCrMo) head articulating with the ultrahigh molecular weight polyethylene (UHMWPE) inner liner. The COR was reduced anatomically.

Postoperatively, there was no infection, dislocation, or implant failure (Figure 3). The FJS was 29 after 2 weeks and 17 after 6 weeks, which was maintained until 2 years after surgery. HHS was 48 after 2 weeks, 87 after 6 weeks, and 97 after 2 years.

Figure 3Case 1 postoperative radiographs (a–c): immediate, 2 weeks, 6 weeks.

**Figure:**
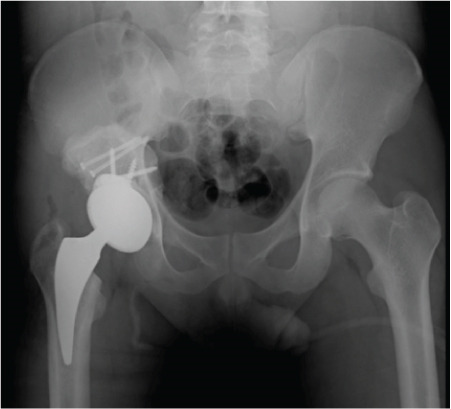
(a)

**Figure:**
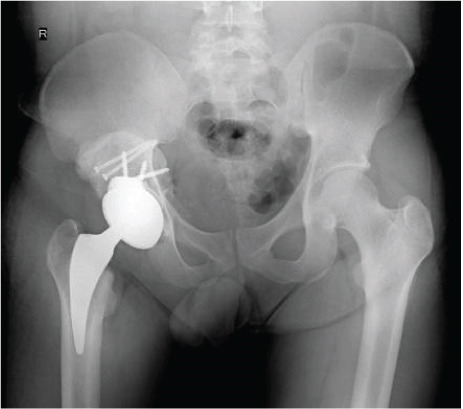
(b)

**Figure:**
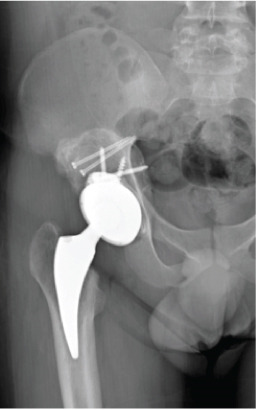
(c)

### 2.2. Case 2

**Figure 4:**
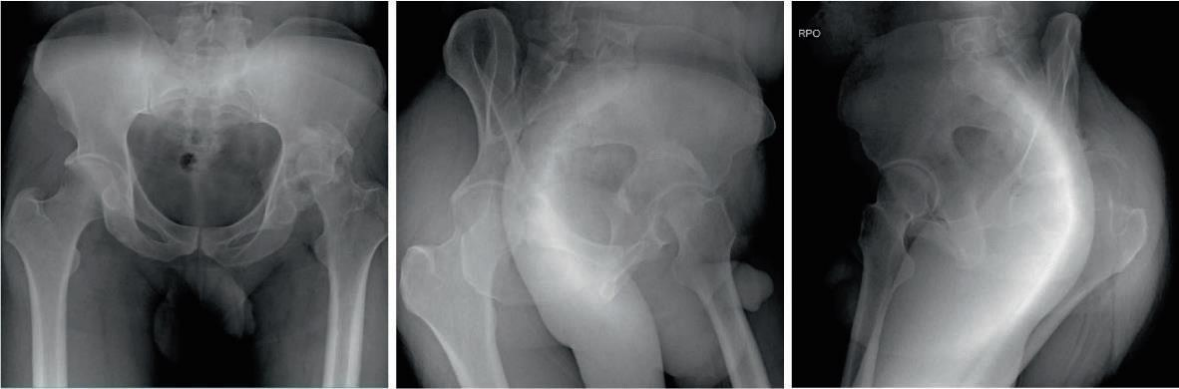
Case 2 preoperative radiographs.

**Figure 5:**
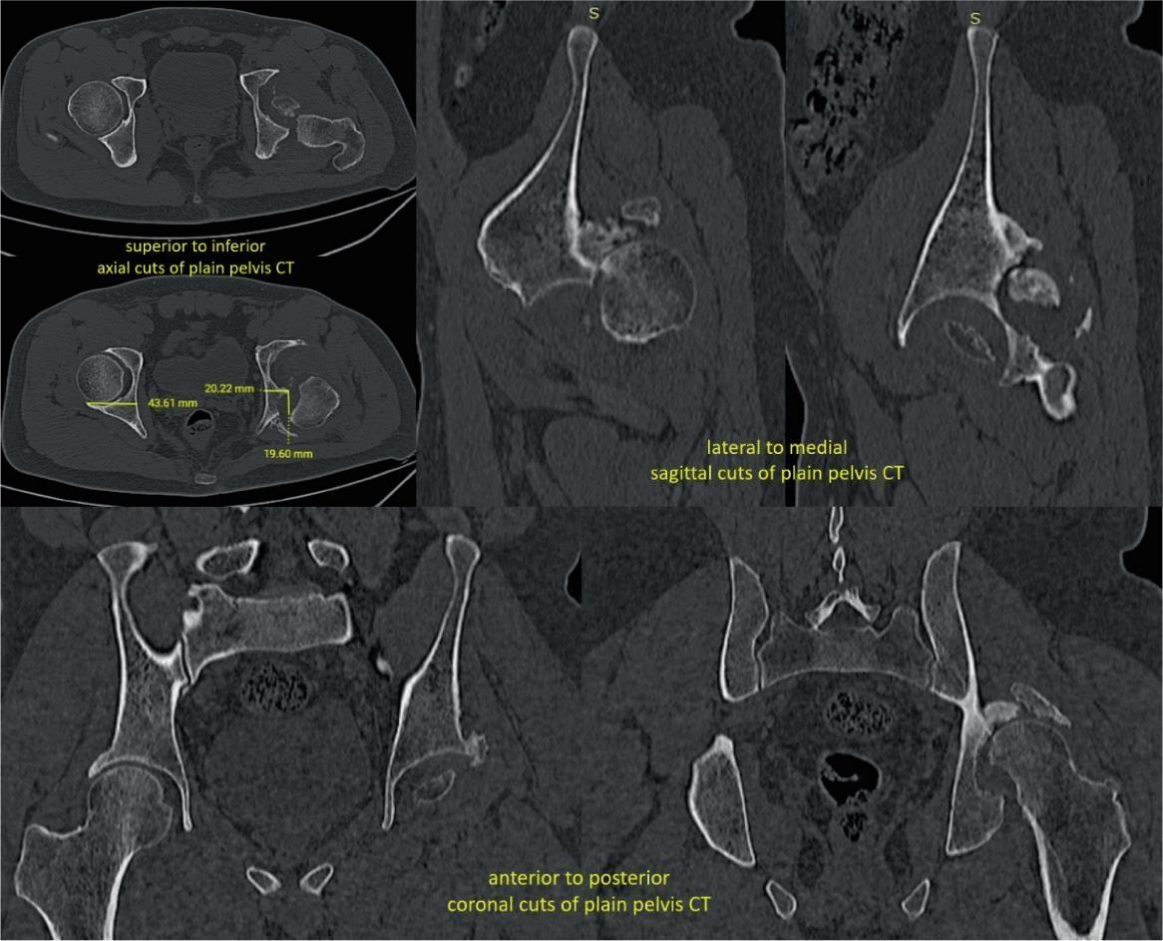
Case 2 preoperative computed tomography.

The second case was a 39‐year‐old male whose posterior wall acetabular fracture was untreated for 32.5 weeks. The fracture left an intra‐articular fragment and a segmental posterosuperior acetabular defect ~2 cm deep involving around 20% of the acetabular rim, classifying the defect as Paprosky IIb. The femoral head had dislocated supero‐posteriorly by 2 cm from the anatomic COR and had created a pseudo‐acetabulum, classifying the dislocation as Thompson and Epstein II. These descriptions can be appreciated in Figures 4 and 5. Preoperatively, the FJS was 25 and the HHS was 57.

A Hardinge approach was utilized to expose the hip then the femoral neck was osteotomized. After medialization and sequential reaming of the acetabulum, the effective segmental defect diminished, and a 48‐mm, cementless, dual mobility cup was seated press‐fit. A Size 4, cementless, short stem and a 22/+3 CoCrMo head articulating with an UHMWPE inner liner were inserted, then the hip was reduced to the anatomic COR.

Postoperatively, FJS was 38 after 2 weeks, 23 after 6 weeks, 16 after 4 months, and 15 after 1 year. HHS was 68 after 2 weeks, 86 after 6 weeks, 95 after 4 months, and 96 after 1 year. There was no incidence of infection, dislocation, or implant failure (Figure 6).

Figure 6Case 2 postoperative radiographs: (a, b) immediate, 2 weeks; (c, d) 6 weeks, 4 months; (e–g) 1 year.

**Figure:**
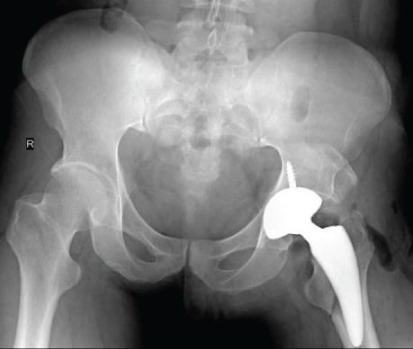
(a)

**Figure:**
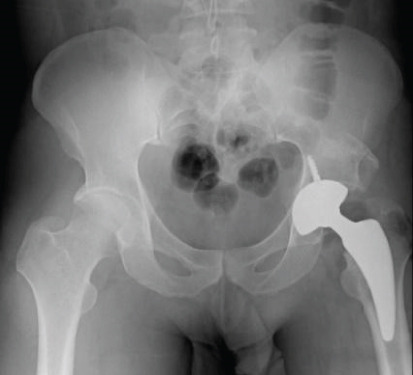
(b)

**Figure:**
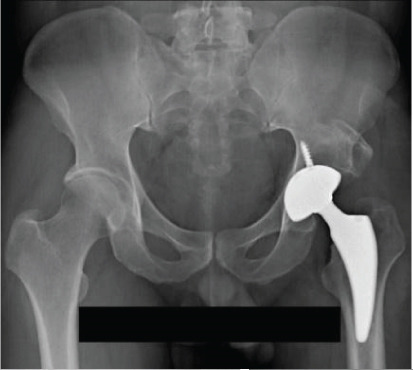
(c)

**Figure:**
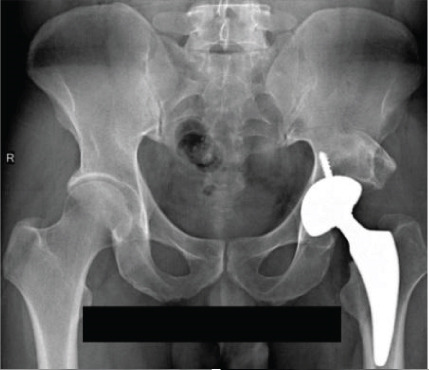
(d)

**Figure:**
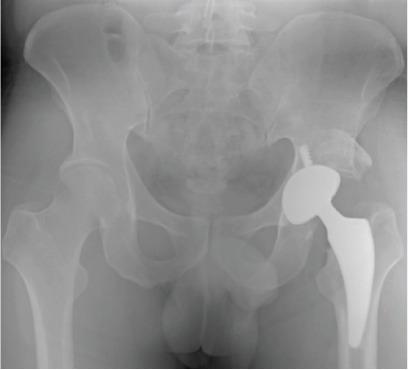
(e)

**Figure:**
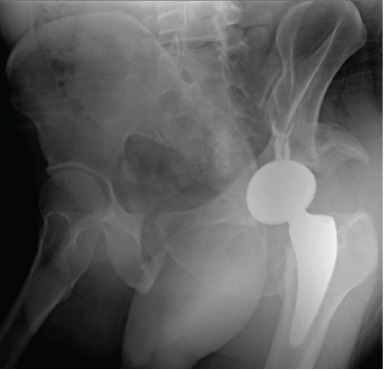
(f)

**Figure:**
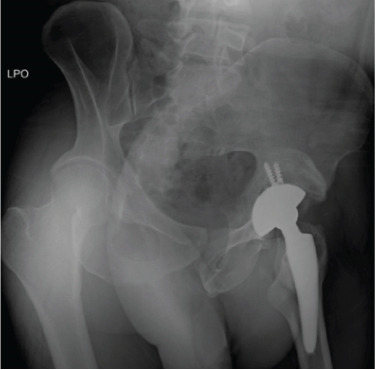
(g)

### 2.3. Case 3

**Figure 7:**
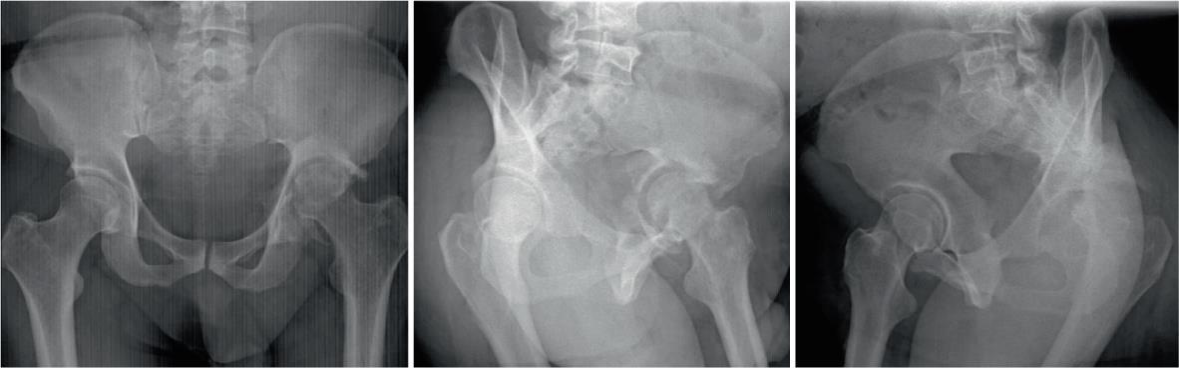
Case 3 preoperative radiographs.

**Figure 8:**
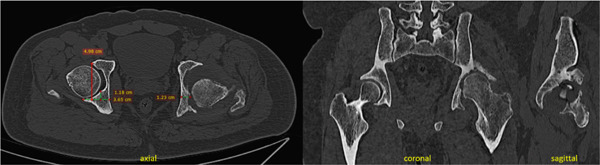
Case 3 preoperative computed tomography.

The third case was a 46‐year‐old male with a posterior wall fracture left untreated for 18.4 weeks. The femoral head had dislocated 1 cm supero‐posteriorly into a pseudo‐acetabulum (Thompson and Epstein III) through a segmental acetabular defect which was around 1 cm deep across about 20% of the posterosuperior rim (Paprosky IIb) (Figures 7 and 8). FJS was 42, and HHS was 65.

A Hardinge approach was used to expose the hip and perform the neck osteotomy. The femoral head was used as a structural bone graft to fill the pseudo‐acetabulum and segmental defect. A 48‐mm, cementless, dual mobility cup was inserted into the anatomic hip COR. The femoral component was composed of a Size 2, cementless, short stem; a 22/0 CoCrMo head; and an UHMWPE inner liner.

Two weeks after surgery, FJS was 19, and HHS was 69. Six weeks after surgery, FJS improved to 16, and HHS was 87. Nine months after surgery, FJS was 13, and HHS was 100. No infection, dislocation, or implant failure occurred during the short‐term postoperative period (Figure 9).

Figure 9Case 3 postoperative radiographs: (a, b) immediate, 2 weeks; (c, d) 6 weeks, 9 months.

**Figure:**
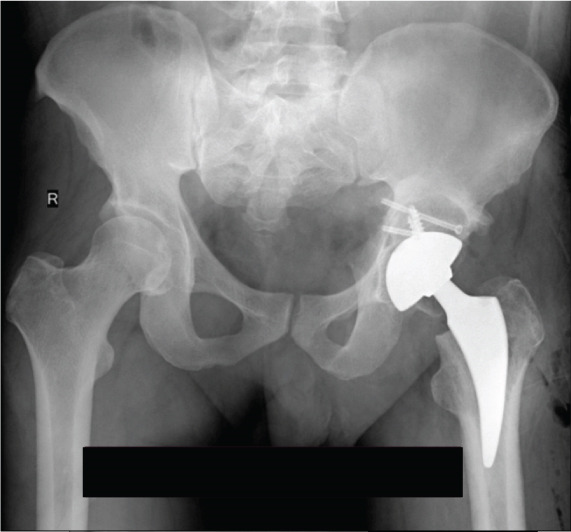
(a)

**Figure:**
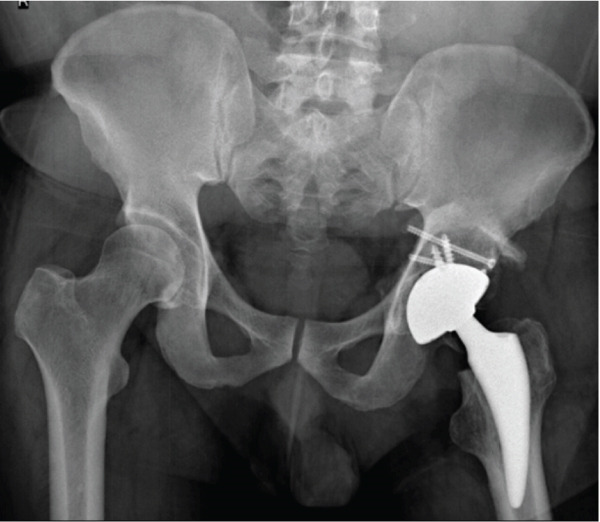
(b)

**Figure:**
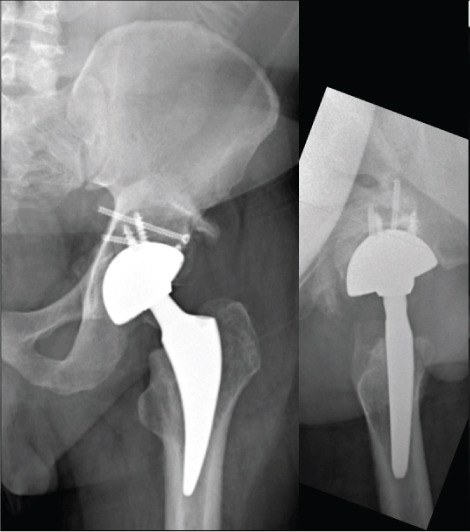
(c)

**Figure:**
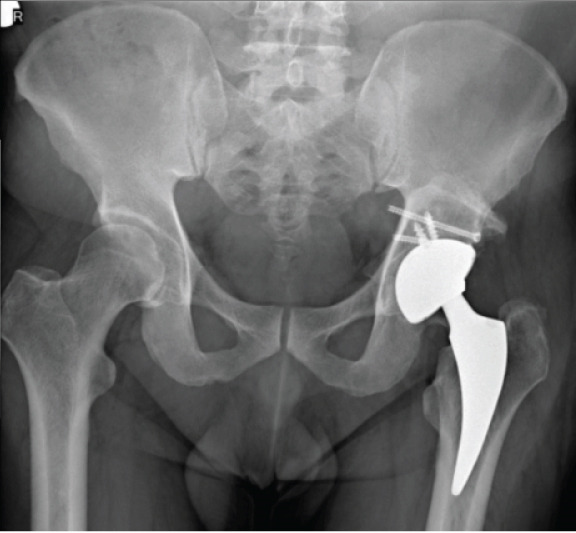
(d)

### 2.4. Case 4

**Figure 10:**
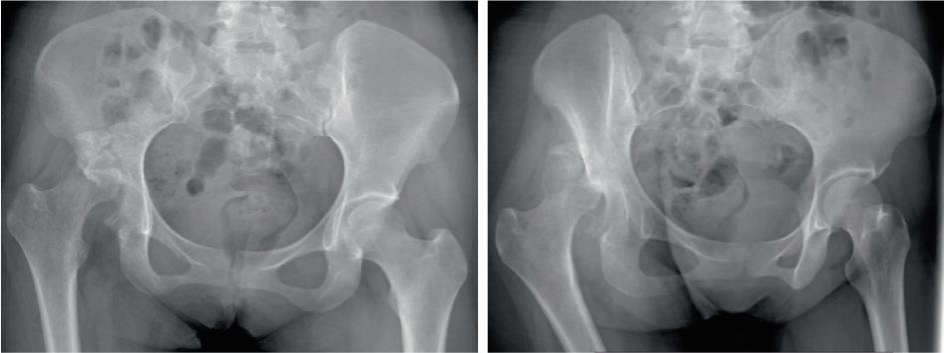
Case 4 preoperative radiographs.

**Figure 11:**
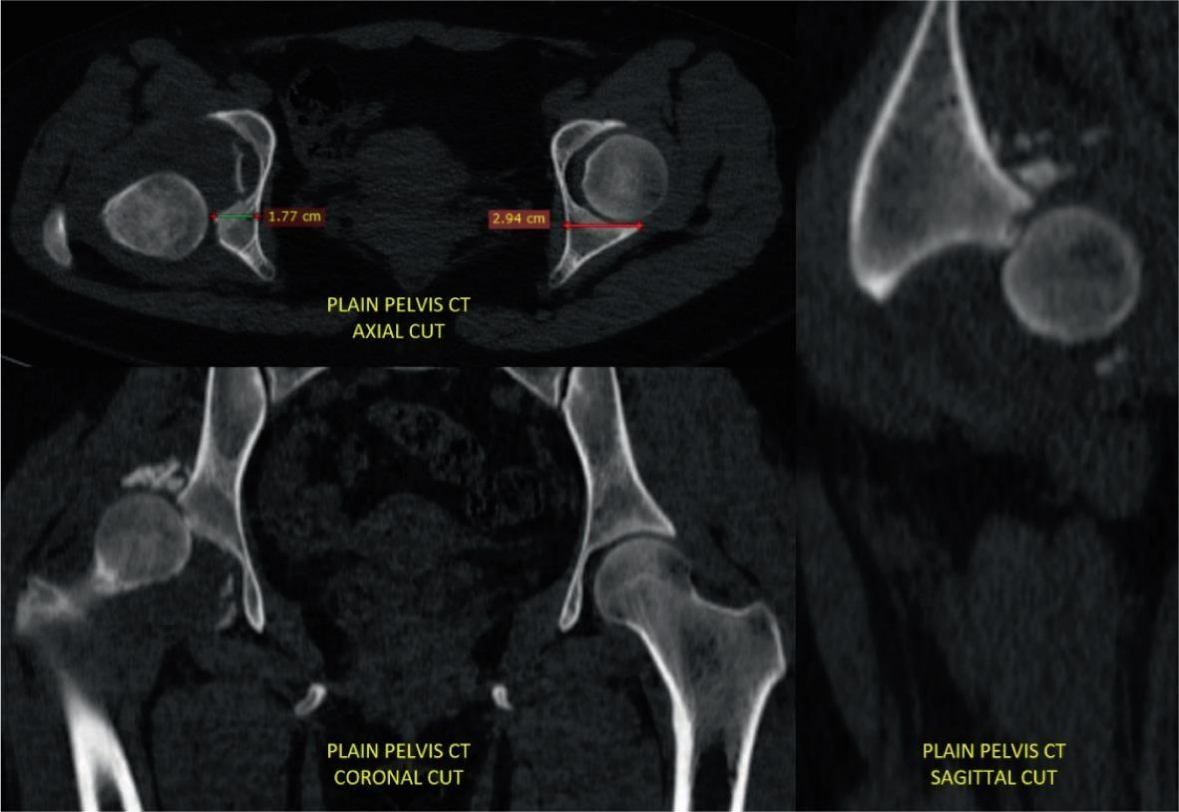
Case 4 preoperative computed tomography.

The fourth case was a 22‐year‐old female with an untreated posterior wall fracture which left an intra‐articular fragment and a posterosuperior, segmental, acetabular defect around 1 cm deep involving ~10% of the acetabular rim classified as Paprosky IIb. The hip was posteriorly dislocated with the COR migrated 2 cm superiorly into a pseudo‐acetabulum classified as Thompson and Epstein III. These findings are shown in Figures 10 and 11. The fracture–dislocation went untreated for 17 weeks. Preoperatively, FJS was 34, and HHS was 51.

The hip was accessed through a Hardinge approach. The femoral head and neck were osteotomized and used as structural bone grafts to fill the pseudo‐acetabulum and acetabular defect. A 50‐mm, cementless, dual mobility cup was placed after medialization and sequential reaming of the true acetabulum. A Size 2, cementless, short stem with a 22/0 CoCrMo head articulating with an UHMWPE inner liner was inserted and reduced into the anatomic COR.

There was no infection, dislocation, or implant failure noted on follow‐up consults (Figure 12). At 2 weeks after surgery, FJS was 30, and HHS was 52. After 2 years, FJS was 12, and HHS was 100.

Figure 12Case 4 postoperative radiographs (a–c): immediate, 2 weeks, 6 months.

**Figure:**
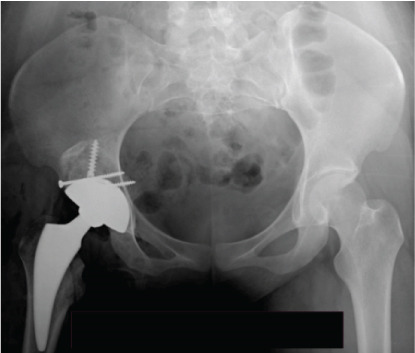
(a)

**Figure:**
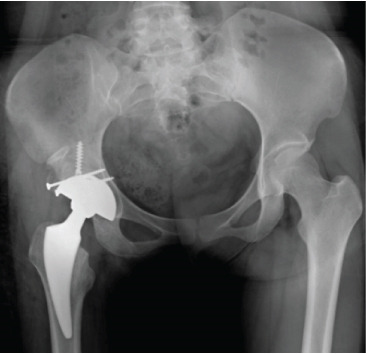
(b)

**Figure:**
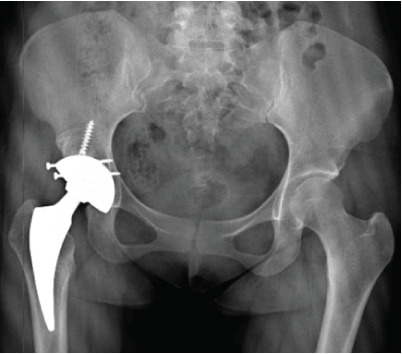
(c)

### 2.5. Case 5

**Figure 13:**
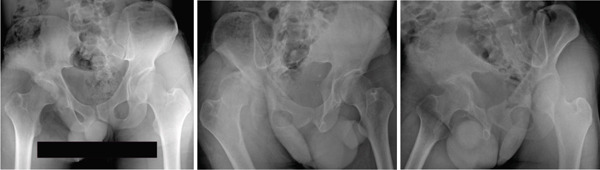
Case 5 preoperative radiographs.

**Figure 14:**
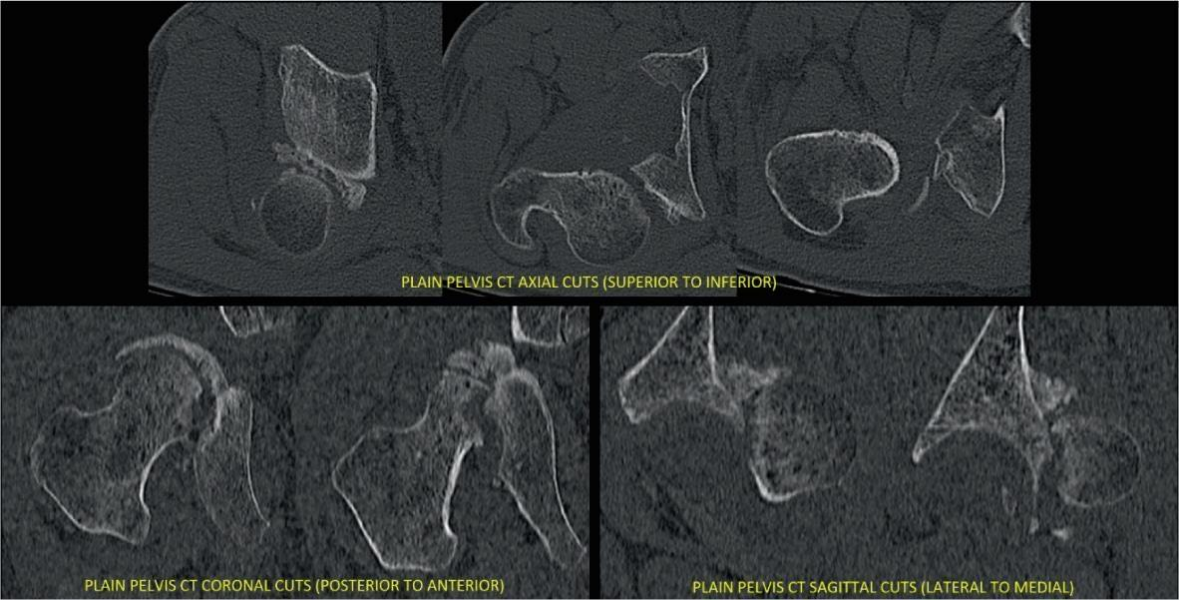
Case 5 preoperative computed tomography.

The fifth case was a 32‐year‐old male with an untreated acetabular fracture at the posterior wall which led to a posterior hip dislocation and superior migration of the COR by 1 cm into a pseudo‐acetabulum which classified the dislocation as Thompson and Epstein III. The resulting posterosuperior segmental defect was < 1 cm and spanned < 10% of the acetabular rim which classified the defect as Paprosky IIb. These findings can be appreciated in Figures 13 and 14. Preoperative FJS was 22, and HHS was 52.

A Hardinge approach was used to expose the hip and perform an osteotomy at the femoral neck. After acetabular reaming, there was adequate coverage for a press‐fit fixation of a 50‐mm, cementless, dual mobility cup. The femoral components used consisted of a Size 4, cementless, short stem and a 22/0 CoCrMo head articulating with a UHMWPE inner liner which allowed the hip to be reduced to its anatomic COR.

Postoperatively, there was no infection, dislocation, or implant failure (Figure 15). After 2 weeks, the FJS was 19, and the HHS was 67. At 4 months after surgery, the FJS was 15 and the HHS was 100.

Figure 15Case 5 postoperative radiographs (a–c): immediate, 2 weeks, 4 months.

**Figure:**
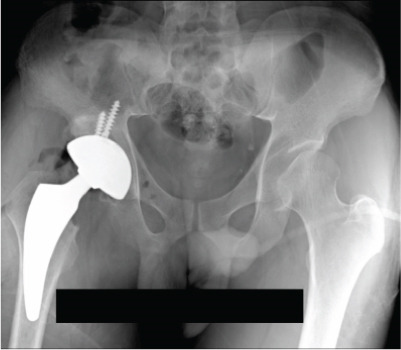
(a)

**Figure:**
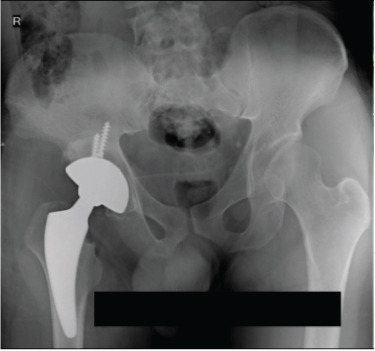
(b)

**Figure:**
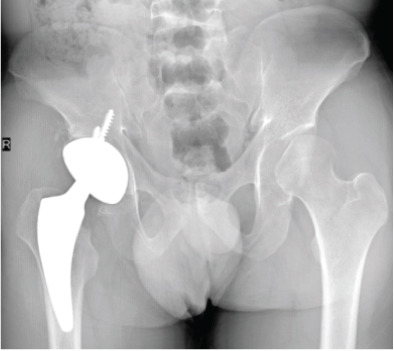
(c)

## 3. Discussion

Acetabular fractures untreated for more than 3 weeks are poor candidates for fixation due to the presence of post‐traumatic hip arthritis and femoral head osteonecrosis [2–4]. This is especially true in cases with poor bone stock, chondral damage, dislocation, comminution, and posterior wall involvement [3, 5]. To address the mentioned complications, total hip arthroplasty is recommended. All five cases in this study presented with post‐traumatic hip arthritis, femoral head osteonecrosis, chondral damage, posterior hip dislocation, and comminuted posterior wall fractures. These characteristics indicated the need for total hip arthroplasty.

The main benefit of total hip arthroplasty in the setting of post‐traumatic arthritis from an acetabular fracture is the postoperative patient‐reported outcome as measured by functional scoring systems [6]. This improvement in symptoms is observed even more than 15 years after surgery [7]. The reported long‐term complications are aseptic loosening of the acetabular component, infection, and prosthesis dislocation, with each having about a 5% occurrence rate [6]. A unique complication in total hip arthroplasty following post‐traumatic arthritis is heterotopic ossification, but this is noted to be asymptomatic [7].

The configurations of the untreated acetabular fractures in this series were assessed using radiographs with pelvis anteroposterior views, Judet views, and hip cross‐table lateral views. The degree of preoperative acetabular bone loss, that is, the extent of the segmental defects, was measured on plain pelvis computed tomography scans with 3D reconstruction. These were also helpful in visualizing intra‐articular fragments. Measurements were taken by the researcher for the purpose of this study. The acetabular defects were described using the Paprosky classification system which correlates the location and the extent of the defect with the support the remaining native bone can give to a standard acetabular cup [8]. The hip dislocations were described using the Thompson–Epstein classification system which mentions the presence of associated hip fractures which may warrant additional interventions [9].

The reconstructive challenges of untreated acetabular fractures include (a) contracted hip musculature, (b) a segmental acetabular defect, and (c) a proximally migrated hip COR [2]. If the COR did not migrate in the presence of a defect, an acetabular cup will likely remain stable without augmentation [4]. If the COR migrated and the defect is until 30% of the entire acetabulum, a structural bone graft or a metal augment may be used to fill the defect [4]. If the defect is more than 30% or there is pelvic discontinuity, an acetabular cage may be necessary to stabilize the acetabular cup [4]. These challenges must be addressed to prevent loosening of the acetabular cup and dislocation of the femoral component.

Cases 1, 3, and 4 had migrated CORs and posterior acetabular defects less than 30%. Case 1 used a combination of a structural bone graft and a metal augment to fill the defect (Table 2). Cases 3 and 4 had sufficient femoral head size and quality that served as the structural bone graft (Table 2). The 4.0 mm, noncannulated, fully threaded cancellous screws were used to fix the structural bone grafts in the three cases.

**Table 2:** Implant characteristics of the cases.

Case	Acetabular augment	Acetabular cup	Femoral head	Femoral stem
1	Structural autologous bone graft, 54 × 10 − mm metal augment	52‐mm dual mobility	22‐mm metal head	Size 2 Type 2A short stem
2	None	48‐mm dual mobility	22‐mm metal head	Size 4 Type 2A short stem
3	Structural autologous bone graft	48‐mm dual mobility	22‐mm metal head	Size 2 Type 2A short stem
4	Structural autologous bone graft	50‐mm dual mobility	22‐mm metal head	Size 2 Type 2A short stem
5	None	50‐mm dual mobility	22‐mm metal head	Size 4 Type 2A short stem

A hip that has dislocated preoperatively has an increased risk for postoperative dislocation due to contractures of the hip capsule, iliopsoas, and gluteus muscles. To maintain the hip in its anatomic COR, contracted soft tissues need to be released and a dual mobility cup may be used [3]. A dual mobility cup prevents dislocation by increasing the jump distance of the femoral head and allowing a wider range of motion prior to neck–cup impingement [10–12]. In the meta‐analysis of Romagnoli et al., dual mobility cups showed significantly reduced dislocation and revision rates compared with standard fixed bearing cups [13]. Since all six cases presented with preoperative posterior hip dislocation, they all employed dual mobility cups to reduce the risk of postoperative dislocation (Table 2).

When performing THA in patients < 55 years old, femoral short stems can be used to preserve the femoral neck and maximize the proximal metaphyseal bone stock [14, 15]. Short stems are proximally loading femoral components less than 120 cm in length, as opposed to conventional stems which load distally and achieve fixation at the diaphyseal canal [14, 15]. The short stems documented in this study were Type 2A (Table 2) which are calcar‐loading and have a trapezoidal cross‐section [16]. By loading at the metaphysis, short stems load at the calcar and prevent postoperative anterior thigh pain unlike in conventional stems [14, 17–19].

Acetabular fractures occur more commonly in the working age group who drive motorcycles. This population has a higher level of activity and a longer projected lifespan which increases their risk for postoperative complications such as infection, dislocation, and implant failure. These must be taken into consideration intraoperatively and monitored on follow‐up consultations.

In this study, none of the cases resulted in postoperative infection, dislocation, or implant failure (Table 3). In the cases documented in this study, FJS improved by 12%–48% and HHS improved by 35%–51% (Table 3). A lower FJS denoted an improvement in patient‐reported outcome as the patient “forgets” or becomes less aware of the previously bothersome joint [20].

**Table 3:** Postoperative outcomes of the cases.

Case	Follow‐up	Complications	FJS improvement	HHS improvement
1	2.7 years	None	43%	51%
2	1 year	None	15%	38%
3	9 months	None	48%	35%
4	2 years	None	37%	49%
5	4 months	None	12%	48%

## 4. Conclusion

Acetabular fractures untreated for more than 3 weeks are predisposed to post‐traumatic arthritis and osteonecrosis of the hip. Internal fixation alone is inadequate to address these complications; hence a total hip arthroplasty allows a better quality of life. Such fractures may present with segmental acetabular defects and chronic hip dislocations. These can be managed with acetabular augments and dual mobility cups. When these injuries present in the young, short stems allow preservation of the femoral neck and stabilization at the proximal metaphyseal cancellous bone.

In this study, untreated acetabular fractures managed with total hip arthroplasty resulted in no infection, dislocation, or implant failure. Functional scores of the six cases improved after surgery by 12%–48% in FJS and 35%–51% in HHS. The limitation of this study is that only short‐term outcomes have been documented. This study can be improved by continuing to monitor the cases for their long‐term outcomes.

## Funding

No funding was received for this manuscript.

## Conflicts of Interest

The authors declare no conflicts of interest.

## Author Biographies

Dr. Christian Emmanuel M. Fontanilla is a board‐certified Fellow of the Philippine Orthopaedic Association. He completed his residency training in orthopaedics at East Avenue Medical Center and took his fellowship training in adult hip and knee reconstruction at Vicente Sotto Memorial Medical Center.

Dr. John N. Hermosisima is a board‐certified Fellow of the Philippine Orthopaedic Association and a member of the Philippine Hip and Knee Society. He completed his residency training in orthopaedics at Vicente Sotto Memorial Medical Center and took his fellowship training in Adult Reconstructive Surgery at Singapore. Currently, he is the training officer for adult hip and knee reconstruction at Vicente Sotto Memorial Medical Center.

Dr. Kenneth Alexis M. Yap is a board‐certified Fellow of the Philippine Orthopaedic Association and a member of the Philippine Hip and Knee Society. He completed his residency training in orthopaedics at Vicente Sotto Memorial Medical Center and took his fellowship training in adult reconstruction at Chong Hua Hospital. Currently, he is the section head for adult hip and knee reconstruction at Vicente Sotto Memorial Medical Center.

Dr. Phillipe Y. Baclig is a board‐certified Fellow of the Philippine Orthopaedic Association and a member of the Philippine Hip and Knee Society. He completed his residency training in orthopaedics at Vicente Sotto Memorial Medical Center and took his fellowship training in adult reconstructive surgery at Singapore. Currently, he is the Chairman of the Department of Orthopaedics and Traumatology at Vicente Sotto Memorial Medical Center.

## Data Availability

The data that support the findings of this study are available from the corresponding author upon reasonable request.
